# Advantage of extracorporeal membrane oxygenation support during catheter ablation for malignant ventricular tachycardia

**DOI:** 10.1016/j.ijcha.2024.101351

**Published:** 2024-02-01

**Authors:** Naoya Kataoka, Teruhiko Imamura

**Affiliations:** Second Department of Internal Medicine, University of Toyama, Toyama, Japan

To editor

Zhang et al. highlighted the efficacy of their modified extracorporeal membrane oxygenation (ECMO) system in reinstating systemic circulation among patients encountering an electrical storm, consequently achieving heightened electrical stability during catheter ablation for ventricular tachycardia (VT) [1]. However, several pivotal concerns merit discussion.

Primarily, two predominant indications necessitate mechanical circulatory support (MCS) during VT ablation, each delineating distinct clinical scenarios. The first pertains to urgent ablation, essential in patients enduring cardiogenic shock, where MCS proves imperative. Conversely, the second indication involves scheduled ablation in patients exhibiting stable hemodynamics at baseline. To ascertain the VT circuit's determinant, VT induction is essential, although this occasionally compromises hemodynamics, warranting MCS. It appears the authors implemented MCS during scheduled ablation [1]. Previous literature has outlined a relatively heightened incidence of bleeding events during Impella-supported VT ablation [2]. Can the authors juxtapose their findings with these antecedent reports?

The Impella, a recently introduced temporary MCS, prompts inquiry into the rationale underlying the authors' preference for ECMO over Impella [1]. Notably, Impella exclusively supports the left ventricle and might occasionally prove inadequate in maintaining hemodynamics when a considerable decrease in preload occurs owing to right ventricular impairment during VT [3]. ECMO, with its capacity for biventricular support, may thus present an advantage in stabilizing hemodynamics during VT. This assertion finds support in the authors' study, where activation mapping could be successfully executed in most patients, albeit encountering limitations in some cases [1]. Could the authors elucidate the reasons underlying the failure to construct activation mapping in certain individuals?

Multifocal VT often manifests in patients with diminished cardiac function, encompassing those of epicardial origin [3]. Notably, cardiac tamponade stands out as a critical complication during epicardial ablation [2]. How did the efficacy and feasibility of epicardial ablation fare during ECMO support?

## Declaration of competing interest

The authors declare that they have no known competing financial interests or personal relationships that could have appeared to influence the work reported in this paper.
